# Biochemical and biophysical characterization of the OXA-48-like carbapenemase OXA-436

**DOI:** 10.1107/S2053230X21008645

**Published:** 2021-08-31

**Authors:** Bjarte Aarmo Lund, Ane Molden Thomassen, Trine Josefine Warg Carlsen, Hanna-Kirsti Schrøder Leiros

**Affiliations:** a The Norwegian Structural Biology Centre (NorStruct), Department of Chemistry, UiT The Arctic University of Norway, 9037 Tromsø, Norway; b Hylleraas Centre for Quantum Molecular Sciences, Department of Chemistry, UiT The Arctic University of Norway, 9037 Tromsø, Norway

**Keywords:** antibiotic resistance, thermostability, carbapenemase OXA-436, X-ray crystal structure

## Abstract

The crystal structure of the class D β-lactamase OXA-436 was solved to a resolution of 1.80 Å. Higher catalytic rates were found at higher temperatures for the clinically important antibiotic imipenem, indicating better adaptation of OXA-436 to its mesophilic host than OXA-48, which is believed to originate from an environmental source. Furthermore, based on the most populated conformations during 100 ns molecular-dynamics simulations, it is postulated that the modulation of activity involves conformational shifts of the α3–α4 and β5–β6 loops. While these changes overall do not cause clinically significant shifts in the resistance profile, they show that antibiotic-resistance enzymes exist in a continuum. It is believed that these seemingly neutral differences in the sequence exist on a path leading to significant changes in substrate selectivity.

## Abstract 2

Possible mechanisms are demonstrated to explain why the antibiotic-resistance enzyme OXA-436 has higher catalytic rates towards last-resort antibiotics under physiological conditions compared with its more widespread homolog OXA-48.

## 1. Introduction   

Carbapenemases are enzymes that hydrolyze the carbapenem class of β-lactam antibiotics, rendering the antibiotics in­effective against bacteria that carry carbapenemase genes (Bush & Bradford, 2016 ▸). It is worrying when carbapenemase genes are detected in pathogenic bacteria belonging to the Enterobacteriaceae family, such as *Klebsiella pneumoniae*. We have previously described the dissemination of the carba­penemase oxacillinase-436 (OXA-436) in pathogenic strains from multiple Danish hospitals and have shown that the enzyme is a class D carbapenemase (Samuelsen *et al.*, 2018 ▸) similar to OXA-48 in terms of substrate specificity. OXA-436 has also been identified in a *Shewanella putrefaciens* strain from a Pakistani hospital (Potter *et al.*, 2017 ▸), and a likely progenitor for the OXA-436 gene has been found in OXA-535 (Jousset *et al.*, 2018 ▸).

Since OXA-48 was first described as a carbapenemase in 2001, several variants have been found in the clinic, and while OXA-48 continues to be the most widespread of the variants, the others appear to fill different niches (Mairi *et al.*, 2018 ▸). With a sequence identity of 91.3% to OXA-48 (Docquier *et al.*, 2009 ▸), we expected OXA-436 to have similar characteristics to other OXA-48-like enzymes. The crystal structures of other OXA-48-like enzymes such as OXA-163, OXA-181, OXA-232, OXA-245 and OXA-405 have previously been published (Lund *et al.*, 2017 ▸; Stojanoski *et al.*, 2015 ▸). Why the sequence diversity within the OXA-48 like group of β-lactamases is so extensive is an open question. Thus, it would be of interest to identify structural features that functionally differentiate the OXA-48-like enzymes. This paper reports the results obtained in X-ray crystallographic studies together with protein stabil­ity measurements using differential scanning calorimetry as well as steady-state kinetics as a function of temperature. The results show that OXA-436 has a higher enzymatic activity at physiological temperatures than its homolog OXA-48. Furthermore, molecular-dynamics (MD) simulations of acyl complexes with substrates are interpreted to shine light on the differences in substrate selectivity between OXA-436 and its more widespread homolog OXA-48, implicating conformational changes in the α3–α4 and β5–β6 loops [as numbered by Docquier *et al.* (2009 ▸) and shown in Fig. 4].

## 2. Materials and methods   

### 2.1. Macromolecule production   

The gene encoding OXA-436 was amplified from a Danish clinical isolate (Samuelsen *et al.*, 2018 ▸) and was cloned into a pDEST-17 vector using exponential megaprimer cloning (EMP) as described previously for OXA-48 (Lund *et al.*, 2014 ▸, 2016 ▸), including an N-terminal hexahistidine (His) tag and a TEV protease cleavage site followed by residues 23–265. Primers were ordered desalted from Sigma–Aldrich. Megaprimers were prepared by mixing 10 µl 5× Phusion HF buffer, 2.5 µl 100 µ*M* forward and reverse primers (see Supplementary Table S1), 1.5 µl 100% DMSO, 23.5 µl nuclease-free water, 8 µl 13 ng µl^−1^ genomic DNA and 1 µl Phusion DNA polymerase. Double-stranded DNA was denatured at 98°C for 30 s, followed by 30 cycles of denaturation at 98°C for 8 s, annealing at 56°C for 20 s and extension at 72°C for 107 s. The sequence for the TEV protease cleavage site was prepared by annealing and extending the primers (Supplementary Table S3) in an anneal-and-extend PCR program. This reaction consisted of 10 µl 5× Phusion HF buffer, 1 µl dNTPs, 2.5 µl 10 µ*M* primers, 1.5 µl DMSO, 33.5 µl nuclease-free water and 0.5 µl Phusion DNA polymerase. The PCR program consisted of 5 min denaturation at 98°C followed by ten cycles of 98°C for 10 s, 56°C for 30 s and 72°C for 30 s, followed by a final 2 min of annealing at 72°C. A second anneal-and-extend step was performed to combine the OXA-436 megaprimer with the TEV protease cleavage site. Finally, the construct was prepared with a final PCR consisting of 10 µl 5× Phusion HF buffer, 1 µl dNTPs, 2.5 µl EMP reverse primer (Supplementary Table S3), 1 µl 50 ng µl^−1^ pDEST-17 vector, 5 µl 40 ng µl^−1^ OXA-436 annealed megaprimer, 29.5 µl nuclease-free water and 1 µl Phusion DNA polymerase. The PCR program consisted of 30 s denaturation at 98°C, followed by 25 cycles of 98°C for 10 s, 56°C for 30 s and 72°C for 30 s, followed by a final 2 min of annealing at 72°C. The DNA was purified using a Macherey–Nagel Nucleospin PCR cleanup kit and phosphorylated using 0.5 µl 10 000 U ml^−1^ T4 PNK ligase (Promega) for 30 min at 37°C followed by ligation overnight at 4°C using 2 µl T4 ligase (Promega). The prepared plasmid was transformed into in-house XL1-Blue cells by a heat-shock protocol and cultures were grown in LB/Amp from single colonies. DNA was extracted using a Wizard Plus SV miniprep DNA-purification system. The resulting sequence was verified by BigDye 3.1 sequencing.

For expression, 2 µl 80 ng µl^−1^ pDEST17_OXA-436_ was transformed into the in-house strain *Escherichia coli* BL21 DE3 STAR pRARE and plated on LB plates with ampicillin. Precultures were grown overnight containing LB, ampicillin and chloramphenicol. 1 l Terrific Broth (TB) medium was inoculated with 10 ml overnight culture and grown to an OD_600_ of 0.7 at 37°C before induction with 400 m*M* isopropyl β-d-1-thiogalactopyranoside overnight. Frozen and rethawed cells were resuspended in 40 ml buffer *A* (50 m*M* Tris pH 7.2, 50 m*M* potassium sulfate) with one tablet of cOmplete EDTA-free protease inhibitor (Merck) and sonicated. The sonicated sample was clarified by centrifugation (18 000*g* for 40 min). The supernatant was loaded onto a 5 ml HisTrap HP column and eluted over a 14 CV gradient to buffer *B* (buffer *A* with 500 m*M* imidazole). Fractions containing OXA-436 were pooled and the His tag was cleaved off overnight using 10 mg in-house His-tagged TEV protease while being dialyzed against buffer *C* (buffer *A* with 300 m*M* NaCl and 2 m*M* β-mercaptoethanol). Contaminants, uncleaved OXA-436 and TEV protease were bound to the HisTrap column during a second run, and the flowthrough containing cleaved OXA-436 was collected. The pure OXA-436 enzyme was concentrated to 13.8 mg ml^−1^ using an Amicon Ultra-15 10 kDa centrifugal filter unit (Merck).

### 2.2. Differential scanning calorimetry   

Purified OXA-436 was dialyzed against 50 m*M* HEPES pH 7.0 supplemented with 50 m*M* potassium sulfate. Two independent experiments were performed at enzyme concentrations of 20 and 40 µ*M*. The enzyme was filtered and degassed. Temperatures were scanned in the range 293–353 K with a gradient of 1 K min^−1^ using a CSC Nano-Differential Scanning Calorimeter III (N-DSC III) with the pressure kept constant at 3 atm. Melting points were determined using the *NanoAnalyze* 3.6 software (TA Instruments, New Castle, Delaware, USA).

### 2.3. Steady-state enzyme kinetics   

The enzymatic properties of purified OXA-48 (as described in previous work; Lund *et al.*, 2014 ▸) and OXA-436 were investigated with 1 n*M* enzyme against imipenem at concentrations from 5 to 0.4 µ*M* using a Spectramax M2e (Molecular Dimensions) at various temperatures. For OXA-436 the temperatures were in the range 294–312 K, while for OXA-48 temperatures between 277 and 312 K were tested. All kinetic experiments were performed under equivalent conditions using buffers supplemented with fresh bicarbonate to ensure the carboxylation of Lys73. Velocities were fitted against concentrations using nonlinear regression with the Michaelis–Menten equation in *GraphPad Prism* 6.0, and an Arrhenius plot was made by linear regression against the confidence intervals.

### 2.4. Crystallization   

**Figure 1:**
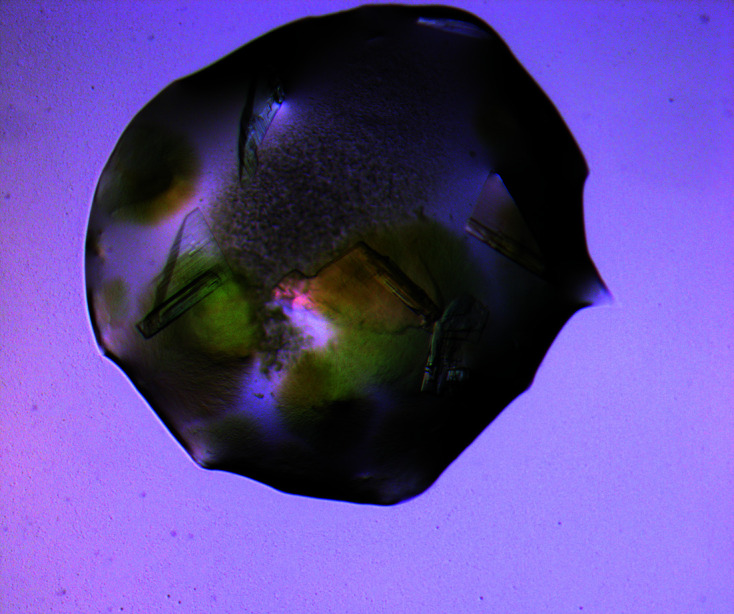
OXA-436 formed plate-like crystals.

Before crystallization, OXA-436 (13.8 mg ml^−1^) was dialyzed into a buffer consisting of 50 m*M* HEPES pH 7.2. A single crystal was observed from condition F2 (25% PEG 3350, 0.2 *M* sodium acetate, 50 m*M* HEPES pH 7.5) of the sparse-matrix SG1 screen (Molecular Dimensions) after incubation at room temperature for three weeks. A plate-like crystal (Fig. 1 ▸) was harvested from a grid screen optimizing the PEG 3350 concentration and the pH. A cryosolution was prepared consisting of 0.1 *M* HEPES pH 8, 25% PEG 3350, 0.2 *M* sodium acetate, 25% ethylene glycol, and the crystal was flash-cooled in liquid nitrogen. Additional crystallization information is summarized in Supplementary Table S2.

### 2.5. X-ray data collection and processing   

Diffraction data were collected on BL14.2 operated by the Joint Berlin MX-Laboratory at the BESSY II electron-storage ring, Berlin-Adlershof, Germany (Mueller *et al.*, 2015 ▸). The images were indexed and integrated using *XDS* (Kabsch, 2010 ▸) and were merged and scaled using *AIMLESS* (Evans & Murshudov, 2013 ▸). X-ray data-collection and processing statistics are summarized in Table 1 ▸.

### 2.6. Structure solution and refinement   

The search model for molecular replacement was prepared from the structure of OXA-48 (PDB entry 5dtk, chains *A* and *D*; Lund *et al.*, 2016 ▸) using *Sculptor* (Bunkóczi & Read, 2011 ▸), and the structure was solved using *Phaser* (McCoy *et al.*, 2007 ▸). Refinement was carried out using *phenix.refine* (Afonine *et al.*, 2012 ▸). The maps were evaluated and the model was manually modified using *Coot* (Emsley *et al.*, 2010 ▸). In the final refinement TLS parameters were refined and refinement weights were optimized. Refinement statistics are summarized in Table 2 ▸.

### 2.7. Molecular-dynamics simulations   

Complexes of OXA-436 with covalently bound ampicillin, imipenem or meropenem were built based on existing OXA-48 complexes and the described OXA-436 structure by aligning the protein coordinates. For ampicillin, the structure of OXA-24 in complex with oxacillin (PDB entry 4f94; C. M. June, B. C. Vallier, R. A. Bonomo, D. A. Leonard & R. A. Powers, unpublished work) was used to build ampicillin manually. For meropenem (PDB entry 6p98; Smith *et al.*, 2019 ▸) and imipenem (PDB entry 5qb4; Akhter *et al.*, 2018 ▸), existing structures with OXA-48 could be used directly. The structures were prepared, energy-minimized and neutralized using sodium ions. The molecular-dynamics simulations were performed in an orthorhombic cell with 10 Å buffer surrounding the monomer (chain *A*). All simulations were of 24 ns after equilibration and used the OPLS3e force field using *Desmond* in *Maestro* 2017.3 (Schrödinger) with default parameters.

## 3. Results and discussion   

**Figure 2:**
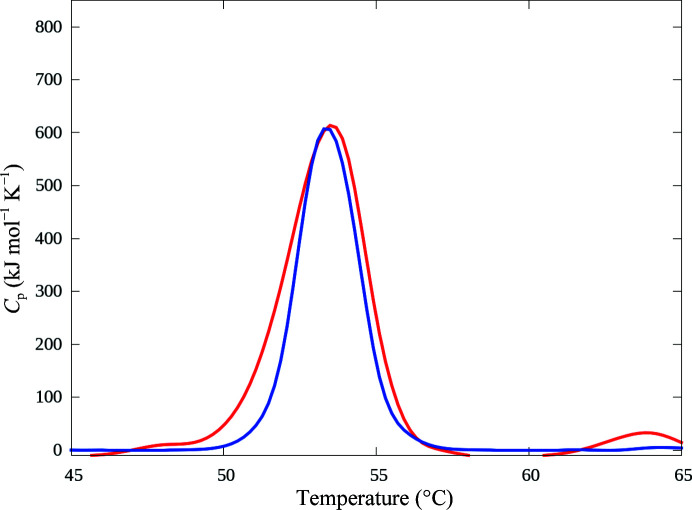
OXA-436 has a melting point of 53.8°C as determined by differential scanning calorimetry; the experimental heating curve shown in red is overlaid with the two-state transition model in blue.

Using differential scanning calorimetry, we determined a melting point of 53.8°C for OXA-436 with a single transition (Fig. 2 ▸), and no refolding was observed after cooling (data not shown). The observed melting point indicates that OXA-436 is less thermostable than OXA-48, which has a melting temp­erature of 55.2°C, but the melting point is in the observed range for other OXA-48-like enzymes (Lund *et al.*, 2017 ▸), and considering the mesophilic environment for the bacterial origin of OXA-436 this is unlikely to be a functional problem.

**Figure 3:**
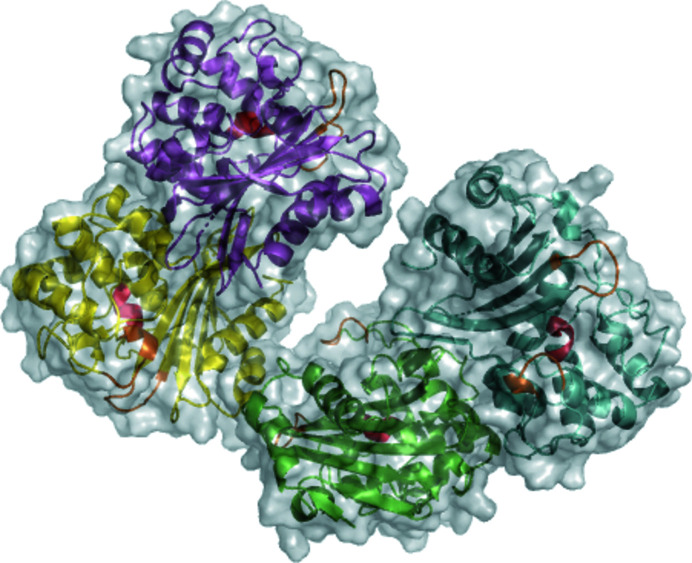
The asymmetric unit of OXA-436 contains four protein chains (*A*–*D*) forming two dimers (chains *A*/*B* in green/blue and chains *C*/*D* in violet/ yellow), where a dimer has been shown to be the biological assembly for close homologs (Lund *et al.*, 2018 ▸). The active sites are independent for each monomer and are marked in red, with the surrounding α3–α4 and β5–β6 loops in orange.

We solved the crystal structure of OXA-436 to high resolution (1.80 Å) with good overall geometry (Davis *et al.*, 2007 ▸). Paired refinement, as outlined by Diederichs & Karplus (2013 ▸) and implemented in the *Phenix* suite (Liebschner *et al.*, 2019 ▸), confirms that the highest resolution shell still contains useful data for refinement with the least-squares target. There are four molecules in the asymmetric unit (chains *A*–*D*; Fig. 3 ▸), and the differences between the chains are localized at surface-exposed polar residues. 1111 water molecules were placed in the electron density, as well as seven molecules of the cryoprotectant ethylene glycol. Chloride ions are observed bridging the dimer interfaces, stabilized by the guanidine group of Arg206 from each chain of the dimers, as has previously been shown for OXA-48 (Lund *et al.*, 2018 ▸), in contrast to the cation-mediated dimerization observed for OXA-10 and OXA-14 (Danel *et al.*, 2001 ▸).

**Figure 4:**
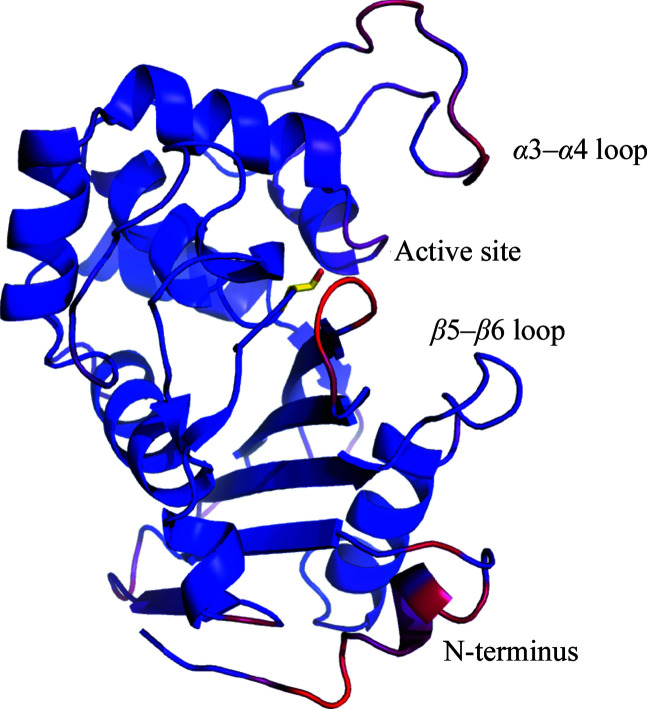
The structure of OXA-436 is coloured according to the C^α^ root-mean-square deviations (r.m.s.d.) compared with OXA-48, with red indicating the maximum deviations. The active-site Ser70 is coloured yellow. The most displaced regions are the N-terminus, the α3–α4 loop and the β5–β6 loop.

Comparison of chain *A* of OXA-436 with OXA-48 (PDB entry 5dtk, chain *A*), to which OXA-436 has 93% sequence identity, gives an r.m.s.d. value of 0.43 Å and a *Q*-score of 0.97 using the protein structure-comparison service *PDBeFold* at the European Bioinformatics Institute (Krissinel & Henrick, 2004 ▸). Based on structural alignment (Fig. 4 ▸), the most significant structural variation appears in the N-terminal part of OXA-436. However, for catalysis, the variations that are observed in the α3–α4 loop and the β5–β6 loop (a sequence alignment is shown in Supplementary Fig. S1) appear to be more interesting. The variations are slight, but from other studies we know that these loops are of high interest (Fröhlich *et al.*, 2021 ▸). Furthermore, there are associated sequence differences in these regions. In the α3–α4 loop there are two differences: Ala104 in OXA-436 corresponds to Thr104 in OXA-48, and Asp110 in OXA-436 is Asn110 in OXA-48. In the β5–β6 loop, there is only a difference between Val213 in OXA-436 and Thr213 in OXA-48 (Supplementary Fig. S1).

### 3.1. Comparing the enzyme kinetics of OXA-436 with OXA-48   

Overall, the enzyme-kinetic parameters of OXA-436 against clinically relevant β-lactam antibiotics are similar to the hydrolytic spectrum found for OXA-48 (Table 3 ▸). This is in line with the expectations based on both gene sequence and structural alignment. Although the differences in hydrolytic properties between OXA-436 and OXA-48 are minor, as shown in Table 3 ▸, we set out to hypothesize how the properties are tuned based on the crystal structure and molecular-dynamics simulations. Based on the known acyl complexes of OXA-48 with imipenem and meropenem and an acyl complex of the structurally similar oxacillin, systems for OXA-48 and OXA-436 were built for the substrates ampicillin, imipenem and meropenem.

**Figure 5:**
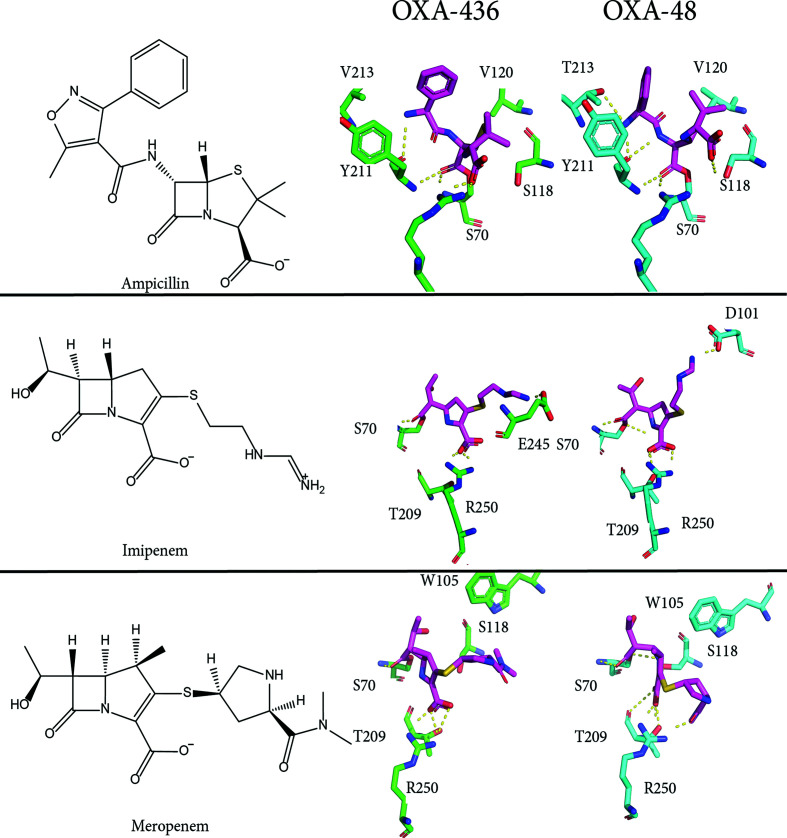
A side-by-side comparison of the primarily polar interactions of OXA-436 (green, middle) and OXA-48 (cyan, right) with the substrates ampicillin, imipenem and meropenem inferred from molecular-dynamics simulations of the acyl complexes reveals differences in preferred binding modes. For each substrate, the molecular structure of the intact antibiotic is shown above the name (left). For meropenem, the tertiary amide in the model with OXA-48 is hidden behind the *R*_2_ substituent carbonyl.

For ampicillin, there is a significant difference in *K*_m_ between OXA-436 and OXA-48. For OXA-436 the *K*_m_ for ampicillin was determined to be 5 µ*M*, whereas for OXA-48 it is estimated to be 395 µ*M*. Thus OXA-436 has a better affinity, with similar values for *k*_cat_, taking the experimental uncertainties into account. Similarly, for imipenem and meropenem there are differences in the Michaelis constants, with similar catalytic efficiencies (*k*_cat_/*K*_m_) for imipenem, but the catalytic efficiency of OXA-436 towards meropenem is fivefold better than that of OXA-48 (Table 3 ▸), which we wanted to explore by molecular dynamics. Based on the determined crystal structures of the acyl complexes, we modelled the corresponding OXA-436 structures and ran molecular-dynamics simulations. From the equilibrated systems, we clustered the most represented conformations for each substrate, as shown in Fig. 5 ▸. In OXA-436, a hydrophobic environment with Val213 and Val120 surrounds the *R*_1_ phenyl ring of ampicillin. This may be more beneficial in stabilizing the acyl complex than the hydrogen bond of Thr213 of OXA-48 to the amino group of ampicillin. A single hydrogen bond contributes on the order of 10 kJ mol^−1^, while the van der Waals interactions stabilizing the hydrophobic interaction are of the order of 4 kJ mol^−1^ per interaction. A well buried phenyl ring has the potential to make several interactions and in this manner can contribute more than a single hydrogen bond. Furthermore, it appears that the differences in the β5–β6 loop to which Val213 belongs would be important for accommodating different *R*_1_ groups. For imipenem, the measured *K*_m_ is higher (lower affinity) for OXA-436 (Table 3 ▸). The ionic interaction of the positively charged *R*_2_ group of imipenem (Skagseth *et al.*, 2016 ▸) with the side chain of Asp101 in OXA-48 is lost in the modelled complex of imipenem with OXA-436 due to movement of the α3–α4 loop. The C^α^ atom of Asp101 is shifted 4.6 Å when comparing the most common cluster for OXA-436 and OXA-48; however, when comparing the crystal structures the difference is only 0.7 Å. The ionic interaction of Asp101 in OXA-48 is only partially replaced by the Glu245 side chain in OXA-436. This movement appears to be induced by the molecular-dynamics simulation relaxing the environment around the bound substrate complex and is of larger magnitude than the subtle differences observed in the crystallo­graphic structure of the apo form. A similar phenomenon was described for the glucosidase MalL, where the structures appeared to be invariant but where local dynamics (as observed by molecular dynamics) led to changes in the enzyme kinetics (Jones *et al.*, 2017 ▸). OXA-436 is observed to have a tighter perceived affinity (*K*_m_) for meropenem than OXA-48. Again, this does not appear to depend on specific polar interactions, but rather the tuning of the active-site cleft by the movement of the α3–α4 loop, which shifts Trp105. There are several differences between the two enzymes, and the observed effects are rather subtle, so it would be unwise to draw strong conclusions from these simulations, especially for enzyme catalysis, which is a complex interplay of reactivity, kinetics and nonbonded interactions. Indeed, the Michaelis–Menten parameters are products of multiple kinetic constants (Galleni & Frère, 2007 ▸), and this complicates comparisons across different enzymes and substrates. However, analysis of the simulations gave several starting points for further mechanistic studies of the subtleties of the carbapenemase activity of the OXA-48-like enzymes, and in the light of Jones *et al.* (2017 ▸), we propose that the sum of the variations between the OXA-436 and OXA-48 enzymes serves to tune the energy landscape for catalysis.

**Figure 6:**
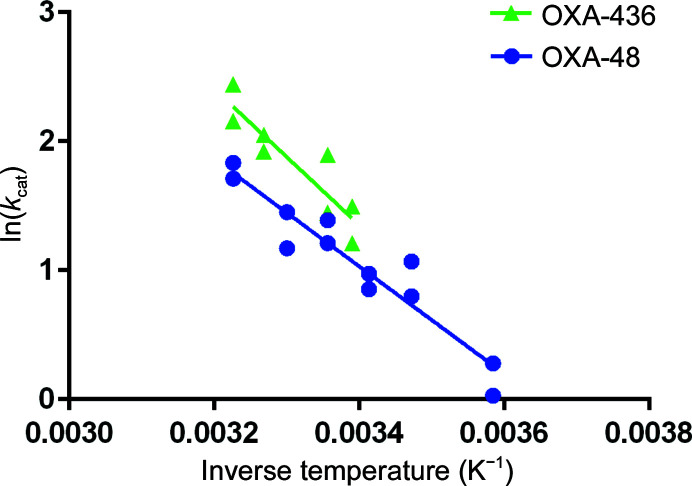
Arrhenius plot of OXA-48 (blue circles) and OXA-436 (green triangles) with the carbapenem substrate imipenem. Symbols indicate the 95% confidence intervals of the determined *k*_cat_ values.

The Arrhenius parameters describe the enzyme activation barrier for the hydrolysis reaction, which using Akaike’s information criteria were shown to be significantly different between OXA-48 and OXA-436 (Table 4 ▸) for the substrate imipenem. Counterintuitively, the measured *k*_cat_ (Table 3 ▸) is nonetheless higher for OXA-436 even though the activation energy is higher when compared with OXA-48, and this is due to the higher pre-exponential factor (*A*) for OXA-436 leading to an intersection between the plots only at subzero temperatures. We demonstrated that OXA-436 has a steeper response to temperature, with higher activities at higher temperatures (Fig. 6 ▸), resembling a human infection scenario, whereas OXA-48 would retain activity at lower temperatures, indicative of an environmental scenario. However, this is not reflected in the antimicrobial susceptibility profiles *in vivo*, where OXA-436 was compared with OXA-48 in genetically identical bacteria, which do not show any significant differences between the two homologs regarding their minimal inhibitory concentrations. This indicates that the sequence differences in OXA-436 alone are not enough to give it a competitive advantage over other OXA-48 variants. We have shown in other work that seemingly neutral sequence changes may lead to dramatic changes in substrate selectivity (Fröhlich *et al.*, 2021 ▸), and variants such as OXA-436 with tuning of the α3–α4 loop and the β5–β6 loop may be a puzzle piece in the big resistance picture.

## 4. Conclusions   

The carbapenem-hydrolyzing class D β-lactamase OXA-436 has been characterized as a mesophilic variant of OXA-48, with an observed average thermal stability of 53.8°C and an increased response to changes in temperature when compared with OXA-48. The observed activation energy towards the carbapenem substrate imipenem was higher for OXA-436 (10.5 kcal mol^−1^) than for OXA-48 (8.3 kcal mol^−1^). From the crystal structure of OXA-436 determined to 1.80 Å resolution, we have used molecular dynamics to explore the differences in substrate hydrolysis, with a particular focus on the α3–α4 loop and the β5–β6 loop as tuneable elements for the substrate selectivity of the OXA-48-like group of enzymes.

The structure of OXA-436 has been deposited in the Protein Data Bank as entry 7oda.

## 5. Related literature   

The following reference is cited in the supporting information for this article: Robert & Gouet (2014 ▸).

## Supplementary Material

PDB reference: OXA-48-like β-lactamase OXA-436, 7oda

Supplementary Figure and Tables. DOI: 10.1107/S2053230X21008645/no5186sup1.pdf

## Figures and Tables

**Table 1:** X-ray data-collection and processing statistics for the OXA-436 crystal structure Values in parentheses are for the outer shell.

Diffraction source	BL14.2, BESSY
Wavelength (Å)	0.91840
Temperature (K)	100
Detector	PILATUS 2M
Crystal-to-detector distance (mm)	328
Rotation range per image (°)	0.1
Total rotation range (°)	180
Exposure time per image (s)	0.3
Space group	*P*2_1_
*a*, *b*, *c* (Å)	69.12, 94.57, 87.76
α, β, γ (°)	90.00, 109.89, 90.00
Resolution range (Å)	44.26–1.80 (1.86–1.80)
Total No. of reflections	324794 (30296)
No. of unique reflections	94891 (9214)
Completeness (%)	95.9 (93.4)
Multiplicity	3.4 (3.3)
〈*I*/σ(*I*)〉	9.46 (1.0)†
*R*_meas_	0.147 (1.26)
*R*_p.i.m._	0.079 (0.67)
CC_1/2_	0.993 (0.478)
Overall *B* factor from Wilson plot (Å^2^)	18.4

† The mean *I*/σ(*I*) falls below 2.0 for reflections above 1.9 Å; however, CC_1/2_ continues to be significant at the 0.1% level to the highest resolution (58.6% at 1.86 Å).

**Table 2:** Structure solution and refinement of the OXA-436 crystal structure Values in parentheses are for the outer shell.

Resolution range (Å)	44.26–1.80 (1.86–1.80)
Completeness (%)	95.90 (93.40)
No. of reflections, working set	94752 (9199)
No. of reflections, test set	4636 (473)
Final *R*_cryst_	0.185 (0.395)
Final *R*_free_	0.226 (0.425)
No. of non-H atoms	
Protein	8013
Ion	2
Ligand	30
Water	1111
Total	9154
R.m.s. deviations	
Bond lengths (Å)	0.013
Angles (°)	1.12
Average *B* factors (Å^2^)	
Overall	21.73
Protein	20.40
Ion	33.23
Ligand	22.62
Water	31.29
Ramachandran plot	
Most favoured (%)	97.85
Allowed (%)	2.15
Disallowed (%)	0.0

**Table 3:** Steady-state enzyme-kinetic parameters of OXA-436 compared with OXA-48 The data are reproduced from Samuelsen *et al.* (2018 ▸). The values for OXA-48 were initially reported by Docquier *et al.* (2009 ▸).

	*K*_m_ (µ*M*)		*k*_cat_ (s^−1^)		*k*_cat_/*K*_m_ (µ*M* s^−1^)	
Substrate	OXA-436	OXA-48	OXA-436	OXA-48	OXA-436	OXA-48
Ampicillin	5	395	600	955	120	2.4
Imipenem	20	13	6	4.8	0.30	0.37
Meropenem	3	11	0.14	0.07	0.05	0.01

**Table 4:** Thermodynamic parameters for the activation barrier for enzymatic hydrolysis of the carbapenem substrate imipenem by OXA-436 and OXA-48

	*E*_a_ (kcal mol^−1^)	ln *A* (s^−1^)	Δ*H* (kcal mol^−1^)	Δ*G* (kcal mol^−1^)	*T*Δ*S* (25°C) (kcal mol^−1^)
OXA-436	10.5	19.3	9.9	16.5	−6.6
OXA-48	8.3	15.2	7.7	16.8	−9.1
