# Supplementary material for: Biochemical and biophysical characterization of the OXA-48-like carbapenemase OXA-436
Source: Acta Crystallogr F Struct Biol Commun. 2021 Aug 31;77(Pt 9):312–8. doi: 10.1107/S2053230X21008645 (PMC8411929; doi:10.1107/S2053230X21008645)
Supplement: Supplementary file 1 [file f-77-00312-sup1.pdf]

Acta Cryst F

STRUCTURAL BIOLOGY
COMMUNICATIONS

Volume 77 (2021)

Supporting information for article:

Biochemical and biophysical characterization of the OXA-48-like carbapenemase OXA-436

Bjarte Aarmo Lund, Ane Molden Thomassen, Trine Josefine Warg Carlsen and Hanna-Kirsti Schrøder Leiros

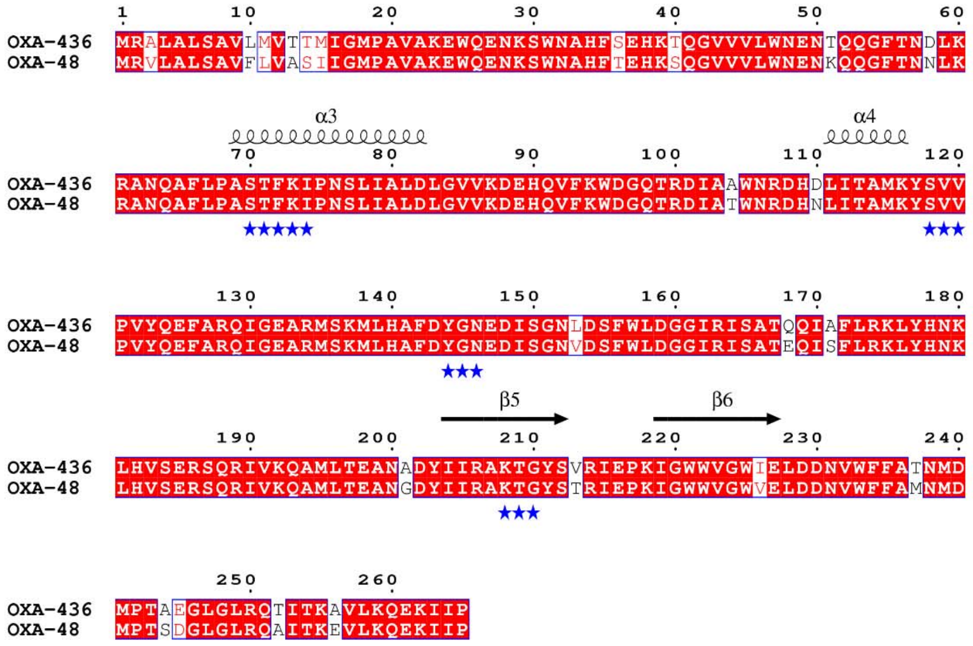

Figure S1 A pairwise sequence alignment of OXA-436 with OXA-48, with selected secondary structure elements numbered according to (Docquier *et al.*, 2009), shows the differences between the two sequences. Selected conserved structural typical to the Oxacillinases are marked with blue stars. The figure was prepared using ESPript (Robert & Gouet, 2014).

Table S1 Macromolecule production information

Source organism	*E. asburiae*
DNA source	Natural
Forward primer	ATAATTTTGTTTAACTTTAAGAAGGAGATATACATATGCGTGCGTTAGCCTTA
Reverse primer	CTTTGTTAGCAGCCTCGAATCACTAAGGAATAATTTTTCCTGTTTCAG
Expression vector	pDEST17
Expression host	*E.coli* BL21 DE3 STAR pRARE
Complete amino acid sequence of the construct produced	*MSYYHHHHHHLESTSLYGENLYFQG*<br>KEWQENKSWNAHFSEHKTQGVVVLWNENTQQGFTNDLK<br>RANQAFLPASTFKIPNSLIALDLGVVKDEHQVFKWDGQTRDIAAWNRDHDLITAMKYSVV<br>PVYQEFARQIGEARMSKMLHAFDYGNEDISGNLDSFWLDGGIRISATQQIAFLRKLYHNK<br>LHVSERSQRIVKQAMLTEANADYIIRAKTGYSVRIEPKIGWWVGWIELDDNVWFFATNMD<br>MPTAEGLGLRQTITKAVLKQEKIIP

The amino acid sequence in italics corresponds to the sequence inserted by the cloning procedure, and includes a TEV protease cleaving site which is used to enzymatically trim the sequence up to the final glycine before the start of the mature OXA-436 protein.

Table S2 :Crystallization parameters yielding OXA-436 crystals suitable for diffraction studies

Method	Hanging drop
Plate type	Hampton Research VDX 24 well
Temperature (K)	294
Protein concentration	13.8 mg/mL
Buffer composition of protein solution	50 mM HEPES pH 7.2
Composition of reservoir solution	0.1 M HEPES pH 7.75, 0.2 M sodium acetate, 26.5% PEG 3350
Volume and ratio of drop	1.5:1.5 µL
Volume of reservoir	1000 µL

Table S3 Primers for plasmid preparation and for exponential amplification

TEV protease site forward	ACCATCACCTCGAATCAACAAGTTTGTACGGTGAGAATCTTTATTTTCAGGGTT
TEV protease site reverse	GGCTTTGTTAGCAGCCTCGAATCAACCCTGAAAATAAAGATTCTCACCG
EMP reverse	TTGTTGATTCGAGGTGATGGTGAT

References

Docquier, J. D., Calderone, V., De Luca, F., Benvenuti, M., Giuliani, F., Bellucci, L., Tafi, A., Nordmann, P., Botta, M., Rossolini, G. M. & Mangani, S. (2009). *Chem Biol* **16**, 540-547.

Robert, X. & Gouet, P. (2014). *Nucleic Acids Research* **42**, W320-W324.
